# Supplementary material for: The Tsallis generalized entropy enhances the interpretation of transcriptomics datasets
Source: PLoS One. 2022 Apr 21;17(4):e0266618. doi: 10.1371/journal.pone.0266618 (PMC9022844; doi:10.1371/journal.pone.0266618)
Supplement: S1 Fig — Dataset is tested for the presence of technical biases using principal variation component analysis (PVCA). Seven possible sources of batch effect were evaluated, in addition to the ‘kinetic’ biological parameter. Residual is the part of the variance not explained by the different parameters tested (A). After normalization, 96.13% of the variance is explained by the kinetic, demonstrating the low impact of technical sources on the dataset (B). (PDF) [file pone.0266618.s001.pdf]

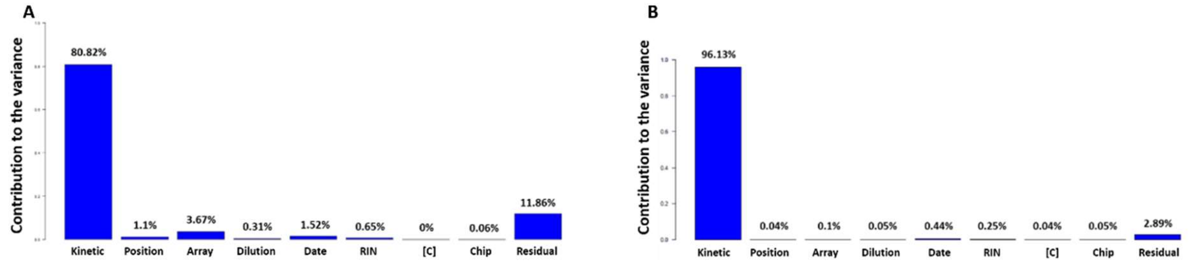

S1 Fig. Data quality by PVCA before and after normalization. Dataset is tested for the presence of technical biases using principal variation component analysis (PVCA). Seven possible sources of batch effect were evaluated, in addition to the 'kinetic' biological parameter. Residual is the part of the variance not explained by the different parameters tested (**A**). After normalization, 96.13% of the variance is explained by the kinetic, demonstrating the low impact of technical sources on the dataset (**B**).
