# Supplementary material for: The Tsallis generalized entropy enhances the interpretation of transcriptomics datasets
Source: PLoS One. 2022 Apr 21;17(4):e0266618. doi: 10.1371/journal.pone.0266618 (PMC9022844; doi:10.1371/journal.pone.0266618)
Supplement: S2 Fig — A: Genes are extracted from day 6 regulated molecular signatures from Nehar-Belaid et al. 2016. B: Genes belonging to both 33 UP and 31 DN gene lists are dispatched based on their eBayes score calculated by comparing gene expression at day 6 to Controls (NP). C: The OTHER subset is created by removing UP and DN subset from the full dataset. Similarly, genes from the 81 down- and 42 up-regulated signatures at day 12 were extracted to build the DN (685 genes), UP (752 genes) and OTHER (10,938 genes) gene lists. (PDF) [file pone.0266618.s002.pdf]

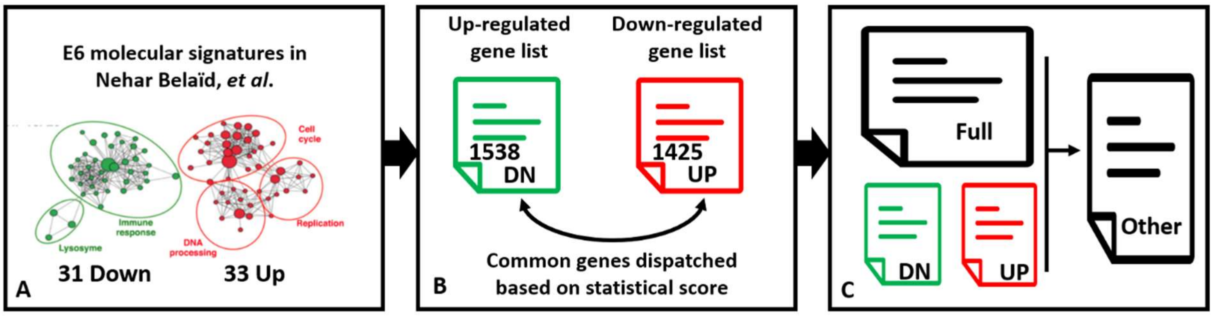

S2 Fig. E6 data subset creation. A: Genes are extracted from day 6 regulated molecular signatures from (Nehar-Belaid et al. 2016). B: Genes belonging to both 33 *UP* and 31 *DN* gene lists are dispatched based on their eBayes score calculated by comparing gene expression at day 6 to Controls (NP). C: The *OTHER* subset is created by removing *UP* and *DN* subset from the full dataset. Similarly, genes from the 81 down- and 42 up-regulated signatures at day 12 were extracted to build the *DN* (685 genes), *UP* (752 genes) and *OTHER* (10,938 genes) gene lists.
