# Supplementary material for: The Tsallis generalized entropy enhances the interpretation of transcriptomics datasets
Source: PLoS One. 2022 Apr 21;17(4):e0266618. doi: 10.1371/journal.pone.0266618 (PMC9022844; doi:10.1371/journal.pone.0266618)
Supplement: S3 Fig — GSEA analysis provided statistical analysis of gene expression biases in the DN (A, D), UP (B, E) and OTHER (C, F) subsets constructed as described in S2 Fig and Material and Methods section. A, E: DN subset shows significant enrichment for down-regulated genes (q-value<10−7). B, E: UP subset shows significant enrichment for up-regulated genes (q-value<10−7). C, F: OTHER subset shows no enrichment (q-value>0.05). (PDF) [file pone.0266618.s003.pdf]

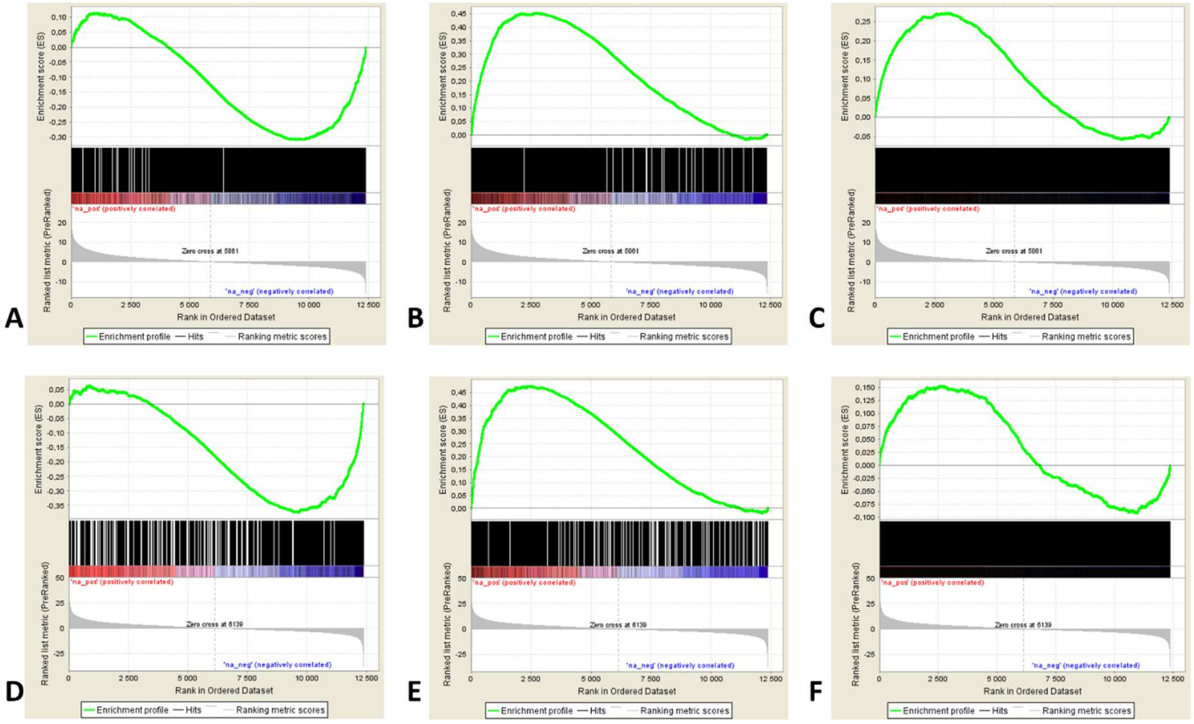


**S3 Fig. Enrichment analysis of day 6 and 12 compared to NP *DN*, *UP* and *OTHER* subsets.** GSEA analysis provided statistical analysis of gene expression biases in the *DN* (**A, D**), *UP* (**B, E**) and *OTHER* (**C, F**) subsets constructed as described in **S2 Fig.** and Material and Methods section. A, E: *DN* subset shows significant enrichment for down-regulated genes (q-value$<10^{-7}$). B, E: *UP* subset shows significant enrichment for up-regulated genes (q-value$<10^{-7}$). C, F: *OTHER* subset shows no enrichment (q-value$>0.05$).
