# Supplementary material for: The Tsallis generalized entropy enhances the interpretation of transcriptomics datasets
Source: PLoS One. 2022 Apr 21;17(4):e0266618. doi: 10.1371/journal.pone.0266618 (PMC9022844; doi:10.1371/journal.pone.0266618)
Supplement: S5 Fig — For each subset, Shannon (A, C and E) and Simpson (B, D and F) alpha diversity were calculated as the average of alpha diversities of individual profiles for the 12/NP DN (A and B), UP (C and D) and OTHER (E and F) subsets. Bars represent the 95% bootstrap confidence interval calculated from 100 iterations where profiles are simulated from a multinomial distribution following the observed transcript frequencies. (PDF) [file pone.0266618.s005.pdf]

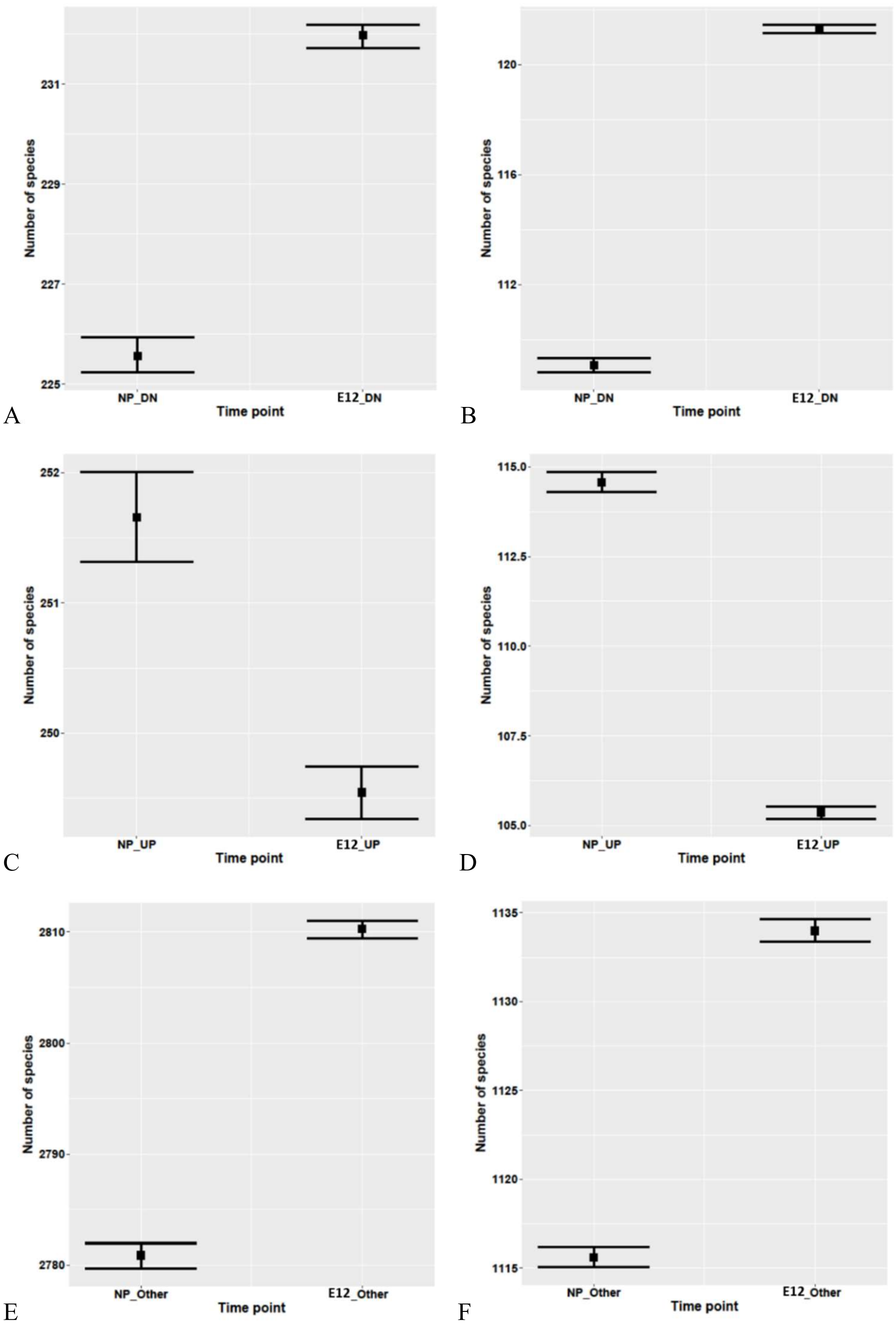

S5 Fig. Alpha diversity analysis of day 12/NP *DN*, *UP* and *OTHER* subsets. For each subset, Shannon (**A, C and E**) and Simpson (**B, D and F**) alpha diversity were calculated as the average of alpha diversities of individual profiles for the 12/NP *DN* (**A and B**), *UP* (**C and D**) and *OTHER* (**E and F**) subsets. Bars represent the 95% bootstrap confidence interval calculated from 100 iterations where profiles are simulated from a multinomial distribution following the observed transcript frequencies.
