# Supplementary material for: The Tsallis generalized entropy enhances the interpretation of transcriptomics datasets
Source: PLoS One. 2022 Apr 21;17(4):e0266618. doi: 10.1371/journal.pone.0266618 (PMC9022844; doi:10.1371/journal.pone.0266618)
Supplement: S1 Graphical abstract — (PDF) [file pone.0266618.s006.pdf]

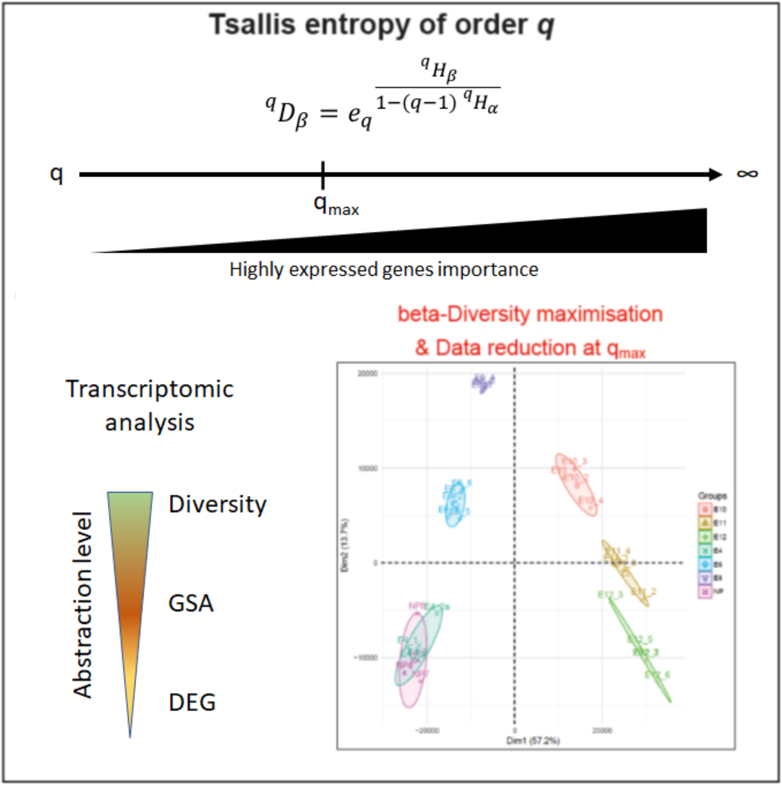
Tsallis entropy of order q
$${}^{q}D_{\beta} = e_{q}^{\frac{{}^{q}H_{\beta}}{1-(q-1)\,{}^{q}H_{\alpha}}}$$
q
q_{max}
∞
Highly expressed genes importance
beta-Diversity maximisation
& Data reduction at q_{max}
Transcriptomic analysis
Abstraction level
Diversity
GSA
DEG
Dim1 (57.2%)
Dim2 (13.7%)
Groups
