# Supplementary material for: The Tsallis generalized entropy enhances the interpretation of transcriptomics datasets
Source: PLoS One. 2022 Apr 21;17(4):e0266618. doi: 10.1371/journal.pone.0266618 (PMC9022844; doi:10.1371/journal.pone.0266618)
Supplement: Attachment — Submitted filename: Derian et al. - Response to reviewers.docx [file pone.0266618.s007.docx]

Derian et al. “The Tsallis generalized entropy enhances the interpretation of transcriptomics datasets”

Point by point response to the reviewers:

Reviewer #1: The paper introduces diversity measures for the analysis of transcriptomes as new tools providing additional information to previous approaches, including a limited use of diversity indices.

It is quite interesting but really hard to follow, so I suggest to make it more straightforward.

My concern is that it focuses so much on the proposed tools that essential aspects of the data are left aside and their very meaning is less clear than in a less sophisticated analysis.
Figure 4A seems to be the basic view of the data. It shows the evolution of the composition of the transcriptome which is very clear with non-overlapping groups except between non pregnant mice and day 4. It should be presented first: diversity measurements only complete this analysis that would be the only one kept if only a very short presentation was allowed.

*We understand the concern of the reviewer, and we thoroughly changed the structure of the manuscript. The first parts of the result section are now focusing on the analysis strategy and data generation aspects. Furthermore, we now present at the beginning of the manuscript (****Figure 1****) the dataset structure using the PCA representation also shown in Figure 4. We believe that this new manuscript structure better presents and outlines the results of our work.*

Instead of that, the paper's organization makes it cryptic. Without going to methods after the conclusion, it is not possible to be sure that individuals are different mice. I strongly suggest a standard presentation with methods before results and data exploration (i.e. fig. 4A) before the proposed new analyses.
*Thank you for the suggestion. We now have moved the material and methods section before the results section and modified it to correct initial ambiguities.*

You must explain clearly what diversity means with respect to the data, not only in general. Here, alpha diversity is that of transcriptomes in an average mouse (strictly speaking, the average diversity of the transcriptomes of the mice of the same group). Beta diversity describes how different from their assemblage these transcriptomes are, i.e. the variability of transcriptomes between mice of the same group.

*We have clarified our definitions of alpha and beta diversities in the context of transcriptomics analyses in the ‘Diversity analysis’ subsection of the materials and methods section (lines 117 on the new manuscript). Additionally, we have carefully edited the manuscript to distinguish ‘transcriptomic profiles’ from ‘biological samples’.*

I'm not convinced by the presentation of beta diversity results.
First, there is a confusion in the introduction of "Exploration of the beta transcriptome diversity indices". Please go back to Jost (2007) cited in Marcon et al. (2014) that you cite. Beta diversity does not capture the variability of alpha diversities. Actually, there is only one alpha diversity that is the average of the diversity of samples. When you write "alpha diversities", you mean "sample diversities". This is not a serious issue but it can be corrected easily.

In the effective number partitioning, gamma diversity is an effective number of transcripts equal to the product of alpha diversity (another effective number of transcripts) and beta diversity, an effective number of samples. Here the sample diversities are exactly the same by definition, and beta diversity is ideally independent from alpha. Beta diversity captures the variability of the composition of samples: how different they are from one another. You write it in line 266.
The confusion arises again line 249: the assemblage gamma diversity is derived from is not the average of samples. Actually, samples diverge from one another (so their assemblage has a higher diversity but this is not the point).

In contrast, the biological interpretation (lines 177-197) is very convincing.

*We thank the reviewer for his careful review of our manuscript. We have now corrected these issues and hope that the new manuscript version meets the reviewer expectations. We also homogenized the vocabulary keeping the term “transcripts abundance” instead of “gene expression level”.*

The method you applied to decomplexify the dataset is a main novelty of the paper. It is not detailed in lines 260-270 so it remains cryptic. It confirms my opinion in changing the order of sections, see above.
*We agree with this suggestion. The section reordering, as stated above, should have clarified this point.*

In the method appendix, it is quite clear. line 414, the meaning of "sum" is not clear. Do you mean "set"?
The interpretation of alpha diversity at q_max is confusing. It is an effective number of genes, i.e. the number of genes equally expressed that would yield the observed diversity. It is not a threshold allowing to detect the most expressed ones: there is probably a very small difference between the D_alpha-th and the D_alpha+1-th gene (ordered from the most to the least expressed). So your choice is reasonable but not obvious. You should make it clearer.

*This is indeed a crucial point: we now better explain these algorithm steps in the Material and Methods (lines 137-154 on the new manuscript) and in the Result section (lines 353-361 on the new manuscript).*

In summary, I recommend major revision. Please rewrite the paper straightforwardly with methods before results, insist less on the novelty of the methods beforehand but be more factual and show that diversity analyses allow to complete previous knowledge (but present it before completing it). You may claim novelty when you interpret the results, showing they were not reachable before.
*We gratefully thank the reviewer for all his constructive remarks. We hope that we have now addressed all of his concerns.*

Minor comments

Please call diversity diversity and entropy entropy. For instance, page 6, paragraph starting line 106: you give the partitioning of entropy in the equation but you write diversity in the first sentence. Then, you write that beta entropy (this is correct) can be transformed into an effective number of samples. This number is beta diversity but the reader is confused by the previous sentence. Line 104, you write that eq.3 describes "beta diversity entropy": this is not correct and very confusing: it describes beta entropy.
*We agree with the reviewer and took care to use, when necessary, the appropriate terms as recommended (e.g., beta diversity definition at lines 216-227 on the new manuscript).*

You can write "diversity of order q": you don't have to refer to entropy, the order applies to both without any ambiguity. e.g., line 125: Increasing the order of diversity gives more weight to highly expressed genes.

*Similarly, this recommendation has been taken into consideration by only referring to the order of the Tsallis entropy function.*

In fig. 1, the way confidence intervals are obtained is not clear.

*We have now specified the way that confidence intervals are obtained (lines 561-562 on the new manuscript). As corrected in the manuscript, ‘Bars represent the 95% bootstrap confidence interval calculated from 100 iterations where profiles are simulated from a multinomial distribution following the observed transcript frequencies’ (as described in the Entropart package). Out of note, this figure corresponds to figure 2 in the new manuscript version.*

Equation 4 is useless: the good practice is to compute beta diversity as the ratio of gamma to alpha since it is easier and allows using a variety of estimators of diversity (out of the scope of the paper).
*Following the suggestion of the reviewer, we have now reformulated equation #3, which takes into consideration the comment of the reviewer about equation #4, see lines 222-227.*

Figure 4: change the labels E4, E6, etc. for E04, E06, etc. so that there are sorted correctly in the legend.
*We now changed the labels of the concerned figures accordingly, which now are figures #1 and #5 in our new manuscript submission.*

Journal Requirements:

When submitting your revision, we need you to address these additional requirements.

1. Please ensure that your manuscript meets PLOS ONE's style requirements, including those for file naming. The PLOS ONE style templates can be found at

https://journals.plos.org/plosone/s/file?id=wjVg/PLOSOne_formatting_sample_main_body.pdf and
https://journals.plos.org/plosone/s/file?id=ba62/PLOSOne_formatting_sample_title_authors_affiliations.pdf

*We have taken this point into consideration and revised the manuscript accordingly.*

2. Thank you for stating the following in the Acknowledgments Section of your manuscript:
"This work has been funded by Assistance Publique-Hôpitaux de Paris, Sorbonne University, Inserm, LabEx Transimmunom (ANR-11-IDEX-0004-02). We thank Wahiba Chaara, Véronique Thomas-Vaslin, Encarnita Ferrandiz for helpful discussions as well as their critical reading of the manuscript"

We note that you have provided funding information that is not currently declared in your Funding Statement. However, funding information should not appear in the Acknowledgments section or other areas of your manuscript. We will only publish funding information present in the Funding Statement section of the online submission form.

Please remove any funding-related text from the manuscript and let us know how you would like to update your Funding Statement. Currently, your Funding Statement reads as follows:

"This work has been funded by Assistance Publique-Hôpitaux de Paris, Sorbonne University, Inserm, LabEx Transimmunom (ANR-11-IDEX-0004-02)"

Please include your amended statements within your cover letter; we will change the online submission form on your behalf

*We have taken this point into consideration and revised the manuscript accordingly. The funding information have been removed from the manuscript.*

3. We note that you have included the phrase “data not shown” in your manuscript. Unfortunately, this does not meet our data sharing requirements. PLOS does not permit references to inaccessible data. We require that authors provide all relevant data within the paper, Supporting Information files, or in an acceptable, public repository. Please add a citation to support this phrase or upload the data that corresponds with these findings to a stable repository (such as Figshare or Dryad) and provide and URLs, DOIs, or accession numbers that may be used to access these data. Or, if the data are not a core part of the research being presented in your study, we ask that you remove the phrase that refers to these data

*We have now removed the references to “data not shown” as we modified the* ***Supplementary Figure 3*** *to include the missing data.*
